# Novel hydrophobic deep eutectic solvent for vortex-assisted liquid phase microextraction of common acaricides in fruit juice followed by HPLC-UV determination

**DOI:** 10.1039/d1ra04781g

**Published:** 2021-09-09

**Authors:** Setareh Rostami-Javanroudi, Masoud Moradi, Kiomars Sharafi, Nazir Fattahi

**Affiliations:** Research Center for Environmental Determinants of Health (RCEDH), Health Institute, Kermanshah University of Medical Sciences Kermanshah Iran n.fattahi@kums.ac.ir +988338263048 +989183364311

## Abstract

In the present research, several novel and natural hydrophobic deep eutectic solvents (DESs) were prepared using methyltrioctylammonium chloride (MTOAC) as the hydrogen bond acceptor (HBA) and different types of straight chain alcohols as hydrogen bond donors (HBDs). One of the DESs composed of MTOAC and *n*-butanol was advantageously used to develop a vortex-assisted liquid phase microextraction (VALPME) method combined with high-performance liquid chromatography-ultraviolet detection (HPLC-UV) for the determination of common acaricides in fruit juice samples. Several important parameters influencing extraction efficiency were investigated and optimized, including the type and volume of DES, sample solution pH, effect of salt addition and, extraction and vortex time. Under optimal experimental conditions, the method showed good linearity with the correlation coefficients (*R*^2^) of 0.9986–0.9991 in the linear range of 2–300 μg L^−1^, low limits of detection of 0.5–1 μg L^−1^ and acceptable extraction recoveries in the range of 85–93%. The proposed method was successfully applied for the extraction and preconcentration of trace acaricides in real fruit juice samples, and the results demonstrated the potential of the synthesized DESs for the extraction and determination of contaminants in aqueous samples.

## Abstract 2

In this research, several novel hydrophobic deep eutectic solvents (DESs) were prepared using methyltrioctylammonium chloride (MTOAC) as the hydrogen bond acceptor (HBA) and different types of straight chain alcohols as hydrogen bond donors (HBDs).

## Graphical abstract

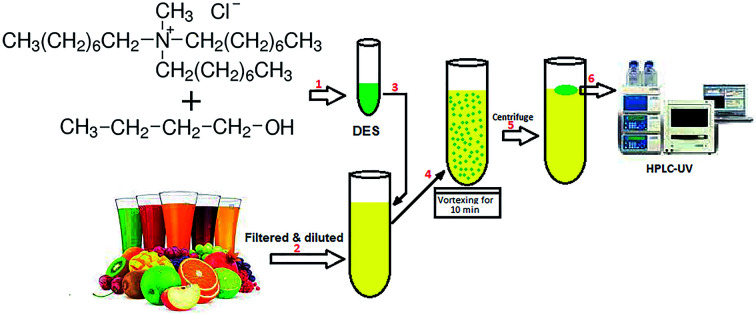

## 1 Introduction

In the present century, many efforts have been made to improve and increase agricultural products. Part of this improvement is related to the use of pesticides to increase agricultural and food production in the world. Clofentezine, fenpyroximate and pyridaben are the most important members of the acaricides family, which are widely used to control insects and mites on vegetables and fruit trees.^1,2^ The use of acaricides is an effective method to control the population of insects and mites and thus increase agricultural products, but the widespread use of these pesticides has led to direct contamination of agricultural and food products and the residue of these toxins in agricultural products and foods, especially fruits and vegetables, have caused public health concerns due to their high consumption in daily life.^3,4^ The European Union (EU) and the Association of the Industry of Juices and Nectars (AIJN) have set a maximum concentration of 0.01 mg kg^−1^ for total pesticides in Directive on Fruit Juice Quality (396/2005/EC).^5,6^

To date, analytical methods for the determination of acaricides in foodstuffs and environmental samples include gas chromatography (GC),^7^ high performance liquid chromatography (HPLC),^8^ liquid chromatography-mass spectrometry (LC-MS)^9^ and gas chromatography-mass spectrometry (LC-MS).^10^ LC-MS and GC-MS are usually employed for determination of acaricides because of high sensitivity, but due to the high cost, the use of these techniques is limited. On the other hand, the HPLC-UV is known to be simple, inexpensive, and found in most laboratories. However, due to the extraction and preconcentration of the samples by the microextraction techniques, acceptable results were obtained by HPLC-UV. Therefore, the sample pre-treatment method is particularly important. It was well known that with the continuous development of technology, in addition to the common solid-phase extraction (SPE) and solid-phase microextraction (SPME) methods, liquid-phase extraction and liquid-phase microextraction (LPME) methods have been popular among researches. In these methods the analyte is extracted into a proper solvent at mL or microliter level.^11^ The used solvent must be immiscible with the sample and form a two-phase system after extraction. Different organic, ionic, and deep eutectic solvents (DESs) have been used in liquid phase extraction and microextraction procedures as the extractive media.^12–21^ In the recent years DESs have attracted many attentions due to their expensiveness, easy preparation, high extraction capability, and less toxicity. DES is a homogeneous and clear solution formed by combining hydrogen bond donor (HBD) with hydrogen bond acceptor (HBA) in a certain ratio at the suitable temperature. Currently, LPME combined with hydrophobic DES as extractant has been suitable for the extraction of different herbicides and pesticides from food and environmental samples.^22–28^

In this study, several novel hydrophobic DESs were synthesized and investigated for the VA-LPME technique of three common acaricides (clofentezine, fenpyroximate and pyridaben) from fruit juice samples in combination with high-performance liquid chromatography coupled with an ultraviolet detection (HPLC-UV). Methyltrioctylammonium chloride (MTOAC) was used as a HBA and ethylene glycol, *n*-butanol, glycerol, *n*-heptanol and *n*-nonanol were used as HBDs. Vortex-assisted was employed to accelerate the dispersion of DES in the aqueous sample for improved extraction recovery and avoid the drawbacks of using disperser solvents. The extraction recovery of the obtained DESs was compared to select the optimum DES, and the main parameters affecting the extraction recovery were optimized, including the volume of the DES, vortex time, sample solution pH and the amount of salt addition. Finally, the proposed hydrophobic deep eutectic solvent for vortex-assisted liquid phase microextraction (DES-VALPME) method was validated under the optimized conditions and employed for the determination of common acaricides in real fruit juice samples.

## 2 Experimental

### 2.1 Reagents and solutions

Clofentezine, fenpyroximate and pyridaben with a certified purity >98% were purchased from Sigma-Aldrich Inc. (St. Louis, MO, USA). Stock standard solutions of clofentezine, fenpyroximate and pyridaben were prepared in methanol (5.0 mL), with concentration of 1000 mg L^−1^ and was stored in a freezer at −20 °C. The working standard solutions were prepared daily by diluting the stock solutions with water to the required concentrations. The ultra-pure water was purchased from Shahid Ghazi Pharmaceutical Co. (Tabriz, Iran). Methanol, acetonitrile, phosphate salt (analytical grade), ethylene glycol, *n*-butanol, glycerol, *n*-heptanol, *n*-nonanol and NaCl were obtained from Merck (Darmstadt, Germany). MTOAC with purity higher than 97% were purchased by Aladdin Biochemical Co., Ltd (Shanghai, China).

### 2.2 Instrumentation

The analysis of target acaricides was achieved on a HPLC Knauer (Berlin, Germany) equipped with a Knauer, Azura UVD 2.1 L UV detector, Azura P 6.1 L pump and a 20 μL injection loop injector (model 7725i, Rheodyne, Cotati, CA, USA). The separation was carried out on an Anachem C18 analytical column (15 cm × 4.6 mm, with 5 μm particle size) from Luton, UK, preceded by a Security Guard Cartridge C18 (Anachem, Luton, UK). The mobile phase consisted of 75% acetonitrile and 25% water at a flow rate of 1.2 mL min^−1^, and the column temperature was maintained at 30 °C. The UV detection wavelength was set to 270 nm for clofentezine and 230 nm for fenpyroximate and pyridaben. Vortex-assisted dispersion of DES was carried out by a vortex shaker Model QL-861 from Haimen, China. The Metrohm pHmeter Model 692 (Herisau, Switzerland) was used for the pH values measurement.

### 2.3 Sampling and sample preparation

Six fruit juice samples including apple, orange, sour cherry, grape, peach and apricot were obtained from local supermarket in Kermanshah, Iran. All fruit juice samples were filtered through 0.22 μm micropore membranes and diluted with ultra-pure water at a ratio of 1 : 1 before performing the extraction method.

### 2.4 DESs preparation

Preparation of the DESs was done by simple mixing of the different organic reagents (ethylene glycol, *n*-butanol, glycerol, *n*-heptanol and *n*-nonanol) as HBD and MTOAC as HBA with a molar ratio of 1 : 1 and the mixtures were heated at 75 °C under magnetic stirring until a transparent and uniform liquid formed. In the following, other molar ratios (3 : 1, 2 : 1, 1 : 2, 1 : 3, 1 : 4, and 1 : 5) of the best DES (MTOAC : *n*-butanol) were also obtained in the same way.

### 2.5 Extraction procedure

A 10 mL of pretreated and diluted fruit juice spiked or not with the target analytes were poured into a glass test tube. Then 100 μL of DES was added, and the extraction was performed by vortexing for 10 min to make the solution turbid. The DES diffused in tiny droplets with a very high contact surface in the sample solution. After centrifugation for 3 min at 5000 rpm, the DES, which also contain analytes slowly accumulate on the surface of the sample solution and float in a droplet. The sample tube was put into ice bath for a 3 min; at this time, the floated DES was solidified because of the low melting point. The solidified DES was transferred into a conical glass test tube where it was melted at room temperature and diluted with the same volume of methanol to reduce viscosity. Finally, 30 μL of the mixture was injected into the HPLC system.

## 3 Results and discussion

### 3.1 Selection of DES type

**Fig. 1:**
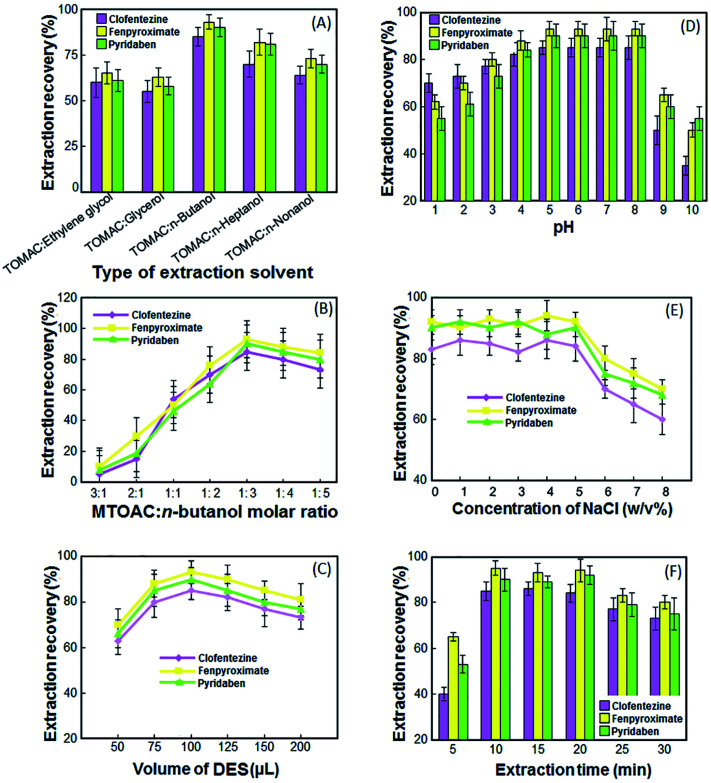
Effect of the different types of extraction solvent (A), molar ratio of MTOAC to *n*-butanol (B), DES volume (C), sample solution pH (D), concentration of NaCl (E) and extraction and vortex time (F) on the extraction recovery of the acaricides in fruit juice. Extraction conditions: types of extractant, MTOAC : *n*-butanol; proportion of MTOAC : *n*-butanol, 1 : 3; volume of the sample solution, 10 mL; sample solution pH, 7; volume of the extraction solvent, 100 μL; extraction and vortex time, 10 min; room temperature.

To effective extraction of the target acaricides from the aqueous solution, DES as an extractant must have a high partition coefficient for the analytes in the extractant, non-volatility and be insoluble in water, and the subsequent determination of the analytes cannot be interfered. Based on these parameters, five DESs including MTOAC : ethylene glycol, MTOAC : *n*-butanol, MTOAC : glycerol, MTOAC : *n*-heptanol and MTOAC : *n*-nonanol were tested as possible extraction solvent. As depicted in Fig. 1A, the obtained results for the extraction recovery (ER%) of the analytes showed that MTOAC : *n*-butanol is more effective that other solvents and it was preferred to use in the next steps.

### 3.2 Selection of MTOAC : *n*-butanol molar ratio

To form a DES, HBD and HBA must be mixed in a certain ratio, and if this ratio is not suitable, a DES will not be formed, or if it is formed, it will have less stability and lower extraction efficiency. In this research, the most suitable molar ratio of MTOAC : *n*-butanol was obtained to achieve best ER. For this purpose, the DESs were obtained by using MTOAC and *n*-butanol with different ratios of 3 : 1, 2 : 1, 1 : 1, 1 : 2, 1 : 3, 1 : 4, and 1 : 5. Experiments in Fig. 1B show that MTOAC and *n*-butanol at a 3 : 1 and 2 : 1 molar ratios could not form DES. The mixture of MTOAC and *n*-butanol in other molar ratios has a positive effect on the ER of the target acaricides. However, DES obtained from a mixture of MTOAC and *n*-butanol in a 1 : 3 molar ratio, has higher ER. So, the 1 : 3 molar ratio of MTOAC and *n*-butanol was chosen for subsequent experiments.

### 3.3 Selection of DES volume

In the present methods, the volume of DES is an important parameter directly affects the ER. On the one hand, insufficient DES volume can lead to incomplete extraction of analytes. On the other hand, an excess volume of DES will reduce the ER due to dilution effect. To investigate the effect of the volume of DES on ER of target analytes, different volumes of selected DES including 50, 75, 100, 125, 150 and 200 μL were employed to perform the experiments under the same conditions. As shown in Fig. 1C, the ER of the target acaricides increased when the volume of DES changed from 50 to 100 μL. When the volume of DES increased from 100 to 200 μL, the ER presented a slightly downtrend. Thus, the maximum ER was obtained when the DES volume was 100 μL.

### 3.4 Selection of sample solution pH

The pH value of sample solutions would change the degree of ionization and speciation of analytes, and further influence the partition coefficient and extraction efficiency of the target compounds. The effect of sample solution pH on the ER of the target analytes was investigated in the range of 1–10. As show in the Fig. 1D, the highest ER was obtained when the pH of the sample phase was between 5–8. This is probably due to the fact that these compounds are ionized at acidic and alkaline pHs due to their molecular structure, and in the range of neutral pHs, their ionization is less and they are more in molecular form. The results demonstrated that the pH value of the samples would not be adjusted for further steps.

### 3.5 Salt effect

For investigating the effect of salt on the extraction recovery of DES-VALPME, various experiments were done by different amounts of NaCl (0–8% NaCl). As shown in the Fig. 1E, with increasing NaCl from 0 to 5%, the ER of target acaricides remain nearly constant, because on the one hand, the salting-out effect increases the ER of target acaricides. On the other hand, the DES solubility in the sample solution decreases and the volume of the collected phase (extractant) increases and due to the dilution effect, the ER of analytes decreases. As a result, these two contrasting effects neutralize each other and the efficiency of target acaricides remains almost constant. At concentrations higher than 5%, the dilution effect prevails on salting-out effect and the ER decreases. Therefore, the experiments were carried out in the absence of any salt.

### 3.6 Effect of extraction and vortex time

In DES-VALPME, extraction and vortex time is defined as the time between injection the DES into the sample solution, and starting to centrifuge. Vortex can assist the dispersion of the DES into the sample solution and speed up the mass transfer of target analytes, which is beneficial to achieve equilibrium faster and enhance the extraction recovery. In this research, the effect of extraction and vortex time was examined in the range of 0–30 min with constant experimental conditions. According to the results presented in Fig. 1F, the highest extraction recovery was reached at 10 min, and there were basically no obvious increasing trends afterwards. In the range of 20–30 min, a small decrease in extraction recovery may be caused by the long-time dispersion of DES, which resulted in the incomplete separation of the two phases after centrifugation.^26^ Therefore, the extraction and vortex time of 10 min was chosen as the optimum time.

### 3.7 Effect of centrifugation speed and time

In this method, the time and speed of the centrifuge are very important in the effective separation of the two phases. In this regard, the centrifugation of samples with different speeds (3000, 4000, 5000, 6000 and 7000 rpm) and different times (1, 2, 3, 4, and 5 min) were tested for achieving better to improve collection of the extraction phase. The results showed that by increasing the centrifuge speed up to 5000 rpm and increasing the centrifuge time up to 3 min, the maximum extraction recovery is obtained for the target analytes. With further increase in centrifuge speed and time, there was not much change in extraction recovery. Thus, the optimum centrifugation speed and time were 5000 rpm and 3 min, respectively.

### 3.8 Method validation

**Fig. 2:**
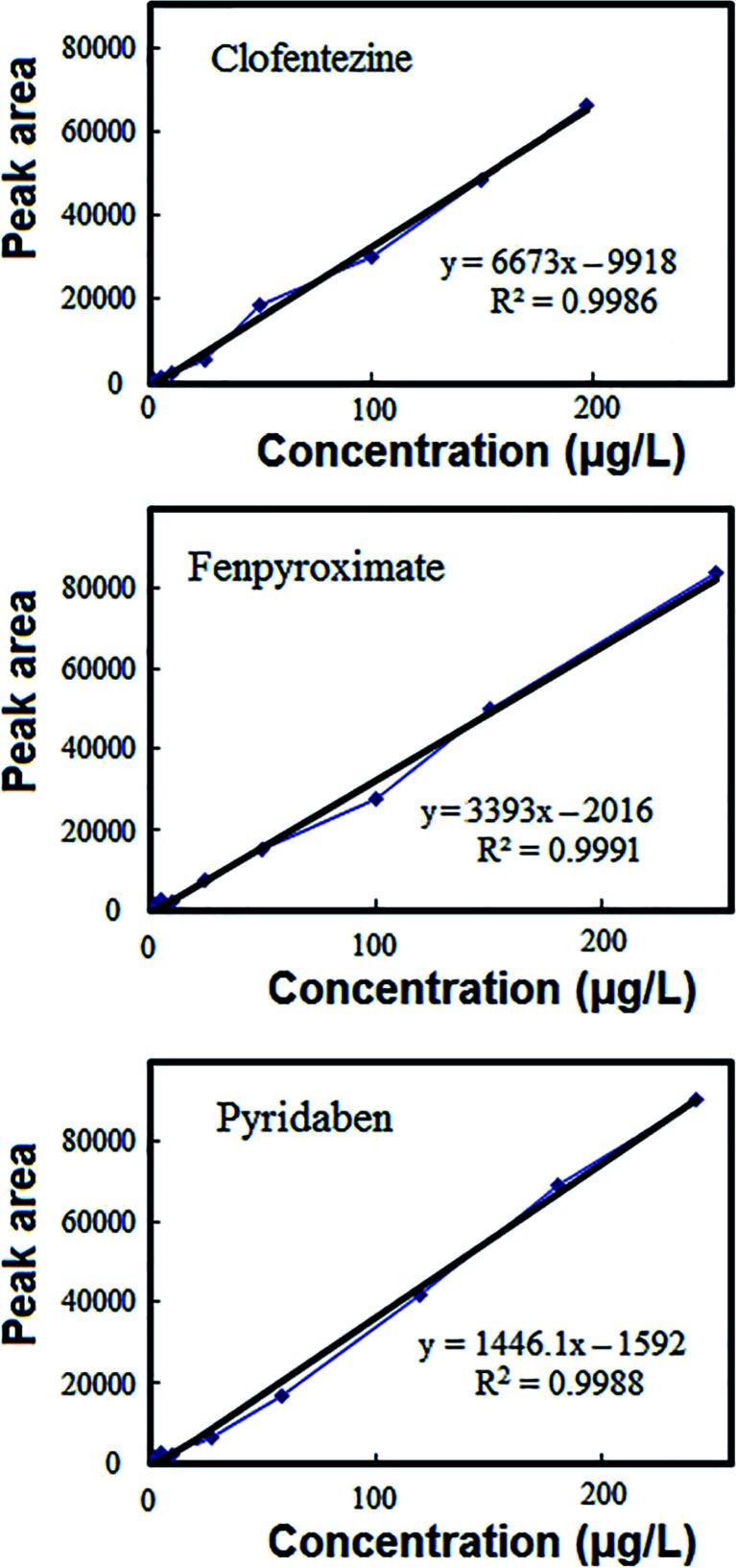
Calibration curves of target acaricides obtained under optimized conditions.

The DES-VALPME method was validated with respect to linearity (LR), limit of quantification (LOQ), limit of detection (LOD), precision including repeatability (intra-day) and reproducibility (inter-day), extraction recovery (ER) and enrichment factor (EF). The calibration curves and the characteristics of these curves shown in Fig. 2 and Table 1, respectively. All the points on the working curves were obtained from the average values of three replicates in spiked samples with different concentrations of the target acaricides. The linear range was 2–300 μg L^−1^ with *r*^2^ ranging from 0.9986 to 0.9991, which showed a good linearity. The LODs (signal-to-noise ratio of 3) and LOQs (signal-to-noise ratio of 10) for the three acaricides were in the range of 0.5–1 μg L^−1^ and 2–3 μg L^−1^ respectively. The method was evaluated for accuracy and precision by analysis of quality control (QC) sample at three concentration levels (5, 50 and 100 μg L^−1^) within the calibration range in fruit juice. The prepared samples were analyzed in 7 replicates on the same day for intra-day, and the same samples were analyzed on three consecutive days, for inter-day. Then the acaricides were extracted using above mentioned procedure and the samples were analyzed by optimized HPLC procedure. The quantity recovered from samples were estimated using respective regression equations. The accuracy was expressed as percent recovery and precision was depicted as percent relative standard deviation. Relative standard deviations (RSDs) including intra-day and inter-day of method based on 7 replicate determinations of acaricides were in the range of 2.5–3.8 and 3.6–5.1%, respectively. The inter-day and intra-day accuracy ranged from 92–107% and 91.5–109, respectively. The EF and ER% of the method were 106–116 and 85–93%, respectively, at the concentration level of 50 μg L^−1^ of target acaricides and the sample volume of 10 mL.

**Table 1:** Quantitative result of DES-VALPME and HPLC-UV of common acaricides from fruit juice

Analyte	ER%	EF	RSD% (intra-day, *n* = 7)	RSD% (inter-day, *n* = 7)	LR (μg L^−1^)	*r* ^2^	LOD (μg L^−1^)	LOQ (μg L^−1^)
Clofentezine	85	106	3.8	5.1	3–200	0.9986	1	3
Fenpyroximate	93	116	2.5	3.6	2–300	0.9991	0.5	2
Pyridaben	90	112	3.2	4.7	2–300	0.9988	0.5	2

### 3.9 Analysis of real samples

**Fig. 3:**
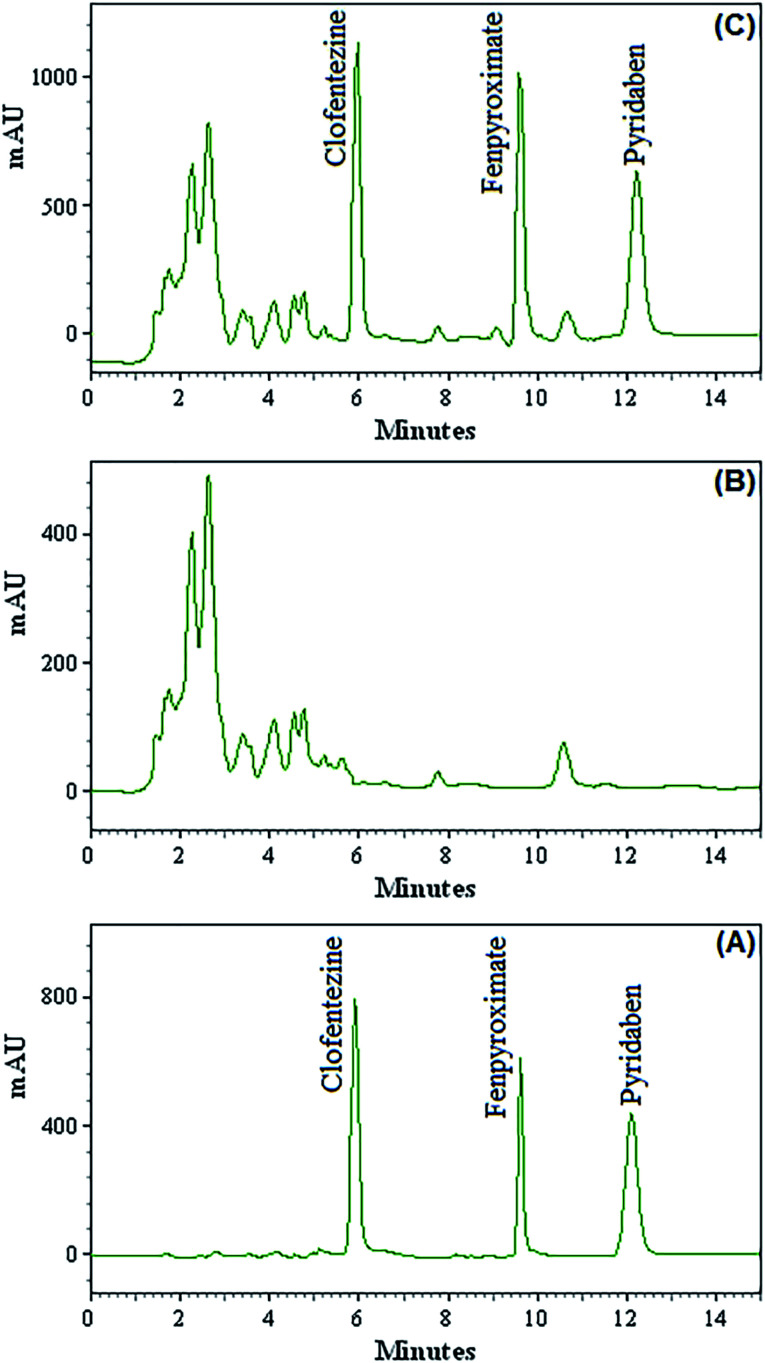
Chromatograms of direct injection of acaricides standards at concentration level of 1.00 mg L^−1^ (A), grape juice sample (B) and the corresponding spiked ones at concentration of 20.0 μg L^−1^ for target acaricides (C) obtained by using DES-VALPME combined HPLC-UV.

The confirm the method applicability in determination of the acaricides in fruit juice, different fruit juice samples including apple, orange, sour cherry, grape, peach and apricot were analyzed by the developed method. The results showed that the analyzed fruit juice samples were free of acaricides contamination. All fruit juice samples were spiked with the standards of three target acaricides at two concentrations (20 and 50 μg L^−1^, each acaricide) to assess matrix effects. The found concentrations were divided to the related spiked values and multiplied to 100 and the obtained ratios are listed as mean relative recoveries in Table 2. Fig. 3 shows the chromatograms of direct injection of target acaricides standards at concentration of 1.00 mg L^−1^ (A), grape juice sample (B) and the corresponding spiked ones at concentration of 20.0 μg L^−1^ for target acaricides (C). Relative recoveries for all acaricides in different samples are between 92–107. These results demonstrate that the fruit juice matrices, in our present context, have no significant effect on DES-VALPME-HPLC-UV for determination of acaricides.

**Table 2:** Relative recoveries and standard deviations of target acaricides from spiked fruit juice samples

Sample	Analyte	Added (μg L^−1^)	Found (mean ± SD) (μg L^−1^)	Relative recovery (%)
Apple juice	Clofentezine	20	19.6 ± 1.2	98
		50	48.8 ± 3.5	97
	Fenpyroximate	20	20.3 ± 0.8	101
		50	49.6 ± 2.7	99
	Pyridaben	20	19.2 ± 0.6	96
		50	51.4 ± 4.1	103
Orange juice	Clofentezine	20	21.2 ± 1.3	106
		50	47.8 ± 3.9	96
	Fenpyroximate	20	18.9 ± 1.8	94
		50	48.6 ± 0.5	97
	Pyridaben	20	20.7 ± 1.3	103
		50	48.0 ± 4.5	96
Sour cherry juice	Clofentezine	20	21.0 ± 1.3	105
		50	53.1 ± 4.0	106
	Fenpyroximate	20	20.8 ± 1.8	104
		50	47.5 ± 2.5	95
	Pyridaben	20	21.2 ± 1.7	106
		50	49.0 ± 3.5	98
Grape juice	Clofentezine	20	20.8 ± 1.5	104
		50	50.8 ± 4.2	102
	Fenpyroximate	20	18.7 ± 1.1	93
		50	47.5 ± 3.3	95
	Pyridaben	20	21.4 ± 2.2	107
		50	48.2 ± 2.7	96
Peach juice	Clofentezine	20	20.5 ± 1.5	102
		50	53.4 ± 4.2	107
	Fenpyroximate	20	19.2 ± 0.7	96
		50	46.6 ± 4.0	93
	Pyridaben	20	18.5 ± 1.7	92
		50	52.8 ± 4.3	106
Apricot juice	Clofentezine	20	21.3 ± 1.2	107
		50	49.0 ± 2.9	98
	Fenpyroximate	20	18.8 ± 1.3	94
		50	47.6 ± 3.3	95
	Pyridaben	20	21.3 ± 1.8	106
		50	51.6 ± 4.7	103

### 3.10 Comparison of DES-VALPME with other methods

The DES-VALPME combined with HPLC-UV is compared with other procedures for determination of acaricides in different samples and the results are summarized in Table 3. As shown in Table 3, the method has the advantage of lower limits of detection as well as a lower extraction time compared to other methods. The consumption of toxic and expensive organic solvents is greatly reduced. The RSD of the presented method are superior to those reported before and the linear range is comparable to other methods and in some cases is better. However, unlike the DLLME method, in this method the disperser solvent is not required. All these results indicate that DES-VALPME is a simple, inexpensive and reproducible technique that can be used for the extraction of acaricides in fruit juice.

**Table 3:** Comparison of DES-VALPME with other extraction methods for determination of acaricides in different samples

Methods	LOD^a^ (μg L^−1^)	LR^b^ (μg L^−1^)	RSD%^c^	Extraction solvent volume	Extraction time (min)	Analytes	Samples	Reference
TEME-HPLC-VWD^d^	21.1–61.4	0.1–600	1.9–3.4	53 μL	16	Chlorfenapyr, fenpyroximate & spirodiclofen	Fruit juice	4
MMHDSPE-HPLC-DAD^e^	0.16–0.57	2.5–5	2	50 mL	30	Clofentezine, fenpyroximate & pyridaben	Fruit juice	8
SPME-GC-MS^f^	2–18	—	7–11	Solvent free	30	Amitraz, bromopropylate, coumaphos and fluvalinate	Honey	10
EA-DLLME-HPLC-DAD^g^	0.07–0.26	1–500	1.22–5.14	100 μL	5	Clofentezine, fenpyroximate, diafenthiuron and pyridaben	Honey	29
SPE-HPLC-UV^h^	1–200	5–800	1.2–7.9	1 mL	30	Flumethrin, chlorfenvinphos, coumaphos, amitraz and cymiazole	Honey	30
LLE-HPLC-DAD^i^	1.5–60 μg kg^−1^	—	1.7–8.8	90 mL	>60	Amitraz, coumaphos, fluvalinate, thymol and rotenone	Honey	31
DLLME-CE^j^	20–57	500–50 000	1.23–5.60	400 + 800 μL	<50	Sulfapyridine, sulfadimidin, sulfadoxin, sulfadiazine and sulfamerazin	Water	32
DES-VALPME-HPLC-UV	0.5–1	2–300	2.5–3.8	100 μL	10	Clofentezine, fenpyroximate & pyridaben	Fruit juice	This work

a LOD, limit of detection.

b LR, linear range.

c RSD, relative standard deviation.

d Totally organic solvent-free emulsification microextraction-high performance liquid chromatography-variable wavelength detector.

e Magnetic mixed hemimicelles dispersive solid-phase extraction-high performance liquid chromatography-diode array detector.

f Solid-phase microextraction-gas chromatography-mass spectrometry.

g Effervescence-assisted, dispersive liquid–liquid mircoextraction-high performance liquid chromatography-diode array detector.

h Solid phase extraction-high performance liquid chromatography-ultraviolet detector.

i Liquid–liquid-extraction-high performance liquid chromatography-diode array detector.

j Dispersive liquid–liquid mircoextraction coupled with capillary electrophoresis.

## 4 Conclusions

In this research for the first time, a novel hydrophobic deep eutectic solvent as extractant for vortex-assisted liquid phase microextraction (DES-VALPME) combined with HPLC-UV has been proposed for the determination of several acaricides in fruit juice samples. In the procedure, a hydrophobic DES consisting of TOMAC as HBD and *n*-butanol as HBA with molar ratio of 1 : 3, was highly effective for extraction and preconcentration of the acaricides. The proposed method can reach equivalent or even higher extraction recovery than the previous methods using conventional organic solvents as extracting agents. The application of this technique in the determination of acaricides in real fruit juice indicated that the proposed method was reliable and suitable for the determination of acaricides in trace levels. Furthermore, the method can be applied to the analysis of target compounds in other complex matrices.

## Conflicts of interest

There are no conflicts to declare.

## Supplementary Material
